# Impact of early PaCO_2_ and pH fluctuations on neurological outcomes in ARDS patients receiving VV ECMO: a retrospective cohort study from the CSECLS registry

**DOI:** 10.1186/s13613-025-01570-9

**Published:** 2025-09-25

**Authors:** Xiaoli Chen, Jinquan Xie, Ya Wang, Zhongtao Du, Xinyi Luo, Zitao Zeng, Haixiu Xie, Jinxiang Ma, Zhangwei Liang, Yin Xi, Jie Zhang, Weibo Liang, Chenglong Li, Zhenting Liang, Jiao Li, Weiqun He, Xiaoqing Liu, Yimin Li, Xiaotong Hou, Yonghao Xu

**Affiliations:** 1 Department of Critical Care Medicine, State Key Laboratory of Respiratory Disease, Guangzhou Institute of Respiratory and Health, Medical Center for Respiratory Medicine, The First Affiliated Hospital of Guangzhou Medical University, Guangzhou, China; 2 Center for Cardiac Intensive Care, Beijing Anzhen Hospital, Capital Medical University, Beijing, China; 3 Department of Critical Care Medicine, The Second Affiliated Hospital of Guangzhou Medical University, Guangzhou, Guangdong China; 4 Department of Statistics, Guangzhou Medical University, Guangzhou, China; 5 Guangzhou National Laboratory, Guangzhou, China

**Keywords:** Acute respiratory distress syndrome, Venovenous extracorporeal membrane oxygenation, Arterial partial pressure of carbon dioxide, Neurological complications

## Abstract

**Background:**

Neurological complications significantly contributed to mortality in patients with acute respiratory distress syndrome (ARDS) supported by venovenous extracorporeal membrane oxygenation (VV ECMO). Early fluctuations in arterial partial pressure of carbon dioxide (PaCO_2_) during ECMO initiation may have affected cerebral perfusion and increased the risk of brain injury. This study investigated the association between early changes in PaCO_2_ and pH levels and subsequent neurological outcomes in patients with ARDS receiving VV ECMO.

**Methods:**

We conducted a retrospective cohort study using data from adult ARDS patients who underwent VV ECMO between January 2018 and December 2022, sourced from the Chinese Society of Extracorporeal Life Support (CSECLS) Registry. Patients were stratified into clusters based on absolute changes in PaCO_2_ and pH using K-means clustering. Logistic regression models and restricted cubic splines were used to evaluate the associations between these clusters and the occurrence of neurological complications, adjusting for potential confounders.

**Results:**

Among 983 patients included, the incidence of neurological complications was 2.95%. Cluster 1, characterized by significant reductions in PaCO_2_ (median: -50 mmHg, relative reduction: -58%), exhibited the highest rate of neurological complications (11.94%). Cluster 3, with substantial increases in pH and minimal reductions in PaCO_2_, showed a relatively lower rate of neurological complications (3.96%), suggesting that PaCO_2_ fluctuations, rather than pH changes, were primarily associated with neurological complications.

**Conclusions:**

Excessive reductions in PaCO_2_ during the early initiation of VV ECMO, rather than pH elevation, were associated with an increased risk of neurological complications in ARDS patients. Close monitoring and management of PaCO_2_ during ECMO initiation may mitigate this risk.

**Supplementary Information:**

The online version contains supplementary material available at 10.1186/s13613-025-01570-9.

## Background

Acute respiratory distress syndrome (ARDS) is a severe clinical condition characterized by severe hypoxemia and bilateral pulmonary infiltrates, often resulting from pneumonia, sepsis, aspiration, or trauma1 [1]. Despite advances in supportive strategies, ARDS has remained associated with high mortality, with pooled estimates ranging from 39% to over 60% in severe cases [2–4].

Extracorporeal membrane oxygenation (ECMO) is a supportive intervention used in cases of refractory ARDS when conventional strategies, such as lung protective ventilation, prone positioning, and neuromuscular blockade, fail to restore adequate gas exchange [4, 5]. However, ECMO is associated with substantial complications. Among these, neurological complications including intracranial hemorrhage, ischemic stroke, seizures, and brain death are particularly devastating, contributing to significant morbidity and mortality [6, 7]. These neurological events, although clinically distinct, often share overlapping and interrelated pathophysiological mechanisms. Key contributing factors include non-physiological cerebral hemodynamics due to extracorporeal circulation, systemic inflammation, vascular endothelial injury, embolic phenomena (such as microthrombi or air emboli), and gas exchange disturbances (e.g., hypoxemia, hyperoxia, hypocapnia, or hypercapnia). These processes may act synergistically: embolism may lead to ischemia, which in turn, in the setting of coagulopathy or endothelial disruption, may evolve into hemorrhagic transformation or provoke seizures [6, 8]. As such, these complications are increasingly understood as elements along a pathophysiological continuum of ECMO-associated neurological injury. Several risk factors have been found to be associated with neurological complications, including platelet count, pre-ECMO pH value, early change of partial pressure of carbon dioxide (PaCO_2_), renal replacement therapy [9–11].

Hypercapnia was frequently observed in ARDS due to increased physiological dead space, ventilation-perfusion mismatch, and the use of low tidal volume ventilation strategies [12, 13]. In patients with pre-existing hypercapnia, ECMO initiation often resulted in substantial and rapid reductions in PaCO_2_ due to its high efficiency in carbon dioxide removal [9, 14]. Luyt et al. were the first to report an association between early reductions in PaCO_2_ and neurological complications during venovenous ECMO (VV ECMO) [14]. This was later supported by Cavayas et al., who analyzed data from the Extracorporeal Life Support Organization (ELSO) registry and reported that a large relative reduction in PaCO_2_ (more than a 50% reduction compared with pre-ECMO levels) was associated with a 1.7-fold increased risk of neurological complications in ECMO-supported patients with respiratory failure [9]. Such abrupt reduction in PaCO_2_ could lead to cerebral vasoconstriction and reduced cerebral blood flow, increasing the risk of brain injury [15, 16].

Several studies have reported that large reductions in PaCO_2_ during the early phase of ECMO support are associated with an increased risk of neurological complications. For example, Cavayas et al. and Hunsicker et al. demonstrated this relationship in VV ECMO cohorts [17, 18]. While Le Guennec et al. report that a rapid decrease in PaCO_2_ following VA ECMO initiation was independently associated with the risk of intracranial hemorrhage [19]. However, a 10-year cohort study by Yu et al. found no significant association between early changes in PaCO_2_ and the development of acute brain injury [20]. These conflicting findings raised the question of whether neurological complications were directly attributable to PaCO_2_ fluctuations or potentially influenced by concurrent physiological changes, such as alterations in systemic pH.

Furthermore, previous study has shown that venoarterial (VA) ECMO is associated with significantly higher rates of neurological complications compared to VV ECMO [21]. Given these considerations, we aimed to investigate the relationship between early changes in PaCO_2_ and pH and the incidence of neurological complications in ARDS patients supported by VV ECMO. A better understanding of these relationships may help optimize early ECMO management strategies and reduce the risk of neurological complications.

## Methods

### Study design and patients

This multicenter retrospective cohort study utilized anonymized data from the Chinese Society of Extracorporeal Life Support (CSECLS) Registry (ClinicalTrials.gov registration number: NCT04158479). The registry is a voluntary, nationwide database that collects standardized information on ECMO utilization, complications, and outcomes from 112 participating adult and pediatric centers across China. Data were entered via an electronic reporting system with built-in validation mechanisms to ensure accuracy and consistency. One of the authors (Y.H. Xu) obtained access to the database and was responsible for data extraction. Ethical approval for the study was obtained from the First Affiliated Hospital of Guangzhou Medical University (Approval No. ES-2025-K024-01). All procedures adhered to the principles outlined in the Declaration of Helsinki, and informed consent was waived due to the study’s retrospective nature.

Adult patients (≥ 18 years) who received VV ECMO for acute respiratory distress syndrome (ARDS) and reported to CSECLS between January 2018 and December 2022 were included. For patients with multiple ECMO episodes, only data from the first episode were analyzed. Patients over 90 years old, those who received ECMO support for less than 24 h, and those missing pre- or post-ECMO arterial blood gas (ABG) data were excluded.

### Covariates and definitions

Baseline data included demographics, clinical diagnoses, ABG parameters, and comorbidities. Comorbidities extracted from CSECLS registry included chronic cardiovascular disease, diabetes, cerebrovascular accident, chronic respiratory disease, chronic kidney disease, malignancies, cirrhosis, and immunocompromised status. All data were collected directly from the CSECLS database and reviewed for completeness and consistency in accordance with predefined data quality standards. In accordance with CSECLS database input rules, ARDS was defined based on the Berlin definition [22]. Chronic cardiovascular disease included hypertension and coronary artery disease. Cerebrovascular accidents were defined as a documented history of intracerebral hemorrhage or ischemic stroke. Chronic respiratory disease included chronic obstructive pulmonary disease, asthma, bronchiolitis, interstitial lung disease and pulmonary tuberculosis. Chronic kidney disease was defined as requiring regular dialysis prior to admission. Malignancies included solid organ carcinomas, leukemia, lymphoma. Immunocompromised status was defined as a history of organ transplantation or autoimmune disease receiving long-term corticosteroid or immunosuppressant therapy, or a diagnosis of acquired immune deficiency syndrome.

### Outcomes

The primary outcome was a composite of neurological complications occurring after ECMO initiation, including intracranial hemorrhage, ischemic stroke, clinical brain death and seizures. Rather than treating these events as isolated entities, we considered them components of a broader ECMO-related neurological injury spectrum, consistent with approaches used in prior study [9]. This integrated approach better captures the complex and multifactorial nature of ECMO-associated neurological injury. The secondary outcomes included the duration of ECMO support, ECMO weaning success, and in-hospital mortality.

In the CSECLS data registration manual of the database, patient neurological complications are defined as follow: brain death refers to the irreversible loss of all functions of the brain and brainstem; seizures are diagnosed based on clinical manifestations or electroencephalography (EEG); hemorrhage and stroke are defined based on the imaging findings of computed tomography (CT) or magnetic resonance imaging (MRI).

### Principal variables of interest

According to CSECLS database entry protocols, ABG values were recorded within 6 h prior to ECMO initiation (pre-ECMO) and at 24 h after ECMO initiation (post-ECMO). Based on these time points, the absolute and relative changes in PaCO_2_ and pH during the early ECMO phase were calculated as follows:

- Absolute Changes:
  - AbsΔCO_2_ = post-ECMO PaCO_2_ − pre-ECMO PaCO_2_ (mmHg).
  - AbsΔpH = post-ECMO pH − pre-ECMO pH.
- Relative Changes:
  - RelΔCO_2_ = AbsΔCO_2_ / pre-ECMO PaCO_2_.
  - RelΔpH = AbsΔpH / pre-ECMO pH.

### Statistical analysis

All statistical analyses were performed using R (version R4.3.1). Categorical variables were presented as counts and percentages (%), while continuous variables were expressed as mean ± standard deviation (SD) for normally distributed data or median with interquartile range (IQR: 25th–75th percentile) for non-normally distributed data. Group comparisons were conducted using the Student’s t-test for parametric variables and the Mann–Whitney U test for non-parametric variables. The chi-square test was used for categorical variables. A two-sided p-value < 0.05 was considered statistically significant. For variables with less than 30% missing data, multiple imputation by chained equations (MICE) was performed with 10 iterations. The imputed datasets were pooled using Rubin’s rules to generate final estimates.

To explore underlying patterns in gas exchange changes, unsupervised machine learning was applied using K-means clustering based on two input variables: absolute changes in PaCO_2_ (AbsΔCO_2_) and pH (AbsΔpH) during the early ECMO phase. Given the use of two continuous variables, at least four distinct patterns were expected. The optimal number of clusters (k = 5) was determined using both the elbow method and silhouette analysis. The elbow method identifies the point at which adding additional clusters yields minimal improvement in within-cluster variance, while silhouette analysis assesses the coherence of each data point within its assigned cluster. Cluster stability assessment was checked using the average Jaccard index on 1000 bootstrap samples [23]. These clusters were subsequently incorporated into downstream regression models to evaluate their associations with neurological complications and clinical outcomes.

Logistic regression models were used to evaluate the associations between clusters and neurological complications. Odds ratios (ORs) and 95% confidence intervals (CIs) were calculated. Confounders were adjusted in a stepwise manner across three models: Model 1 was unadjusted. Model 2 adjusted for demographic and disease severity indicators that differed significantly between clusters, including sex, body mass index (BMI), and pre-ECMO Sequential Organ Failure Assessment (SOFA) score, and age (due to its known association with mortality in ECMO-supported patients) [24]. Model 3 further adjusted for the duration of invasive mechanical ventilation (IMV) prior to ECMO, as well as baseline comorbidities: chronic cardiovascular disease, cerebrovascular accidents, chronic respiratory disease, chronic kidney disease, diabetes, malignancies, immunocompromised status and cirrhosis. These models were also applied to assess the associations between different clusters and in-hospital mortality, as well as between RelΔCO_2_ groups and the risk of neurological complications.

A restricted cubic spline model was used to access the relationship between PaCO_2_ changes and the risk of neurological complications. To further explore this association, subgroup analyses were conducted based on pre-ECMO PaCO_2_ levels, with *hypercapnic* defined as PaCO_2_ ≥ 50mmHg and *non-hypercapnic* as PaCO_2_ < 50mmHg. This stratified approach provided insights into optimal PaCO_2_ adjustment thresholds and therapeutic targets in VV ECMO supported ARDS patients.

## Results

### Demographic characteristics

**Fig. 1:**
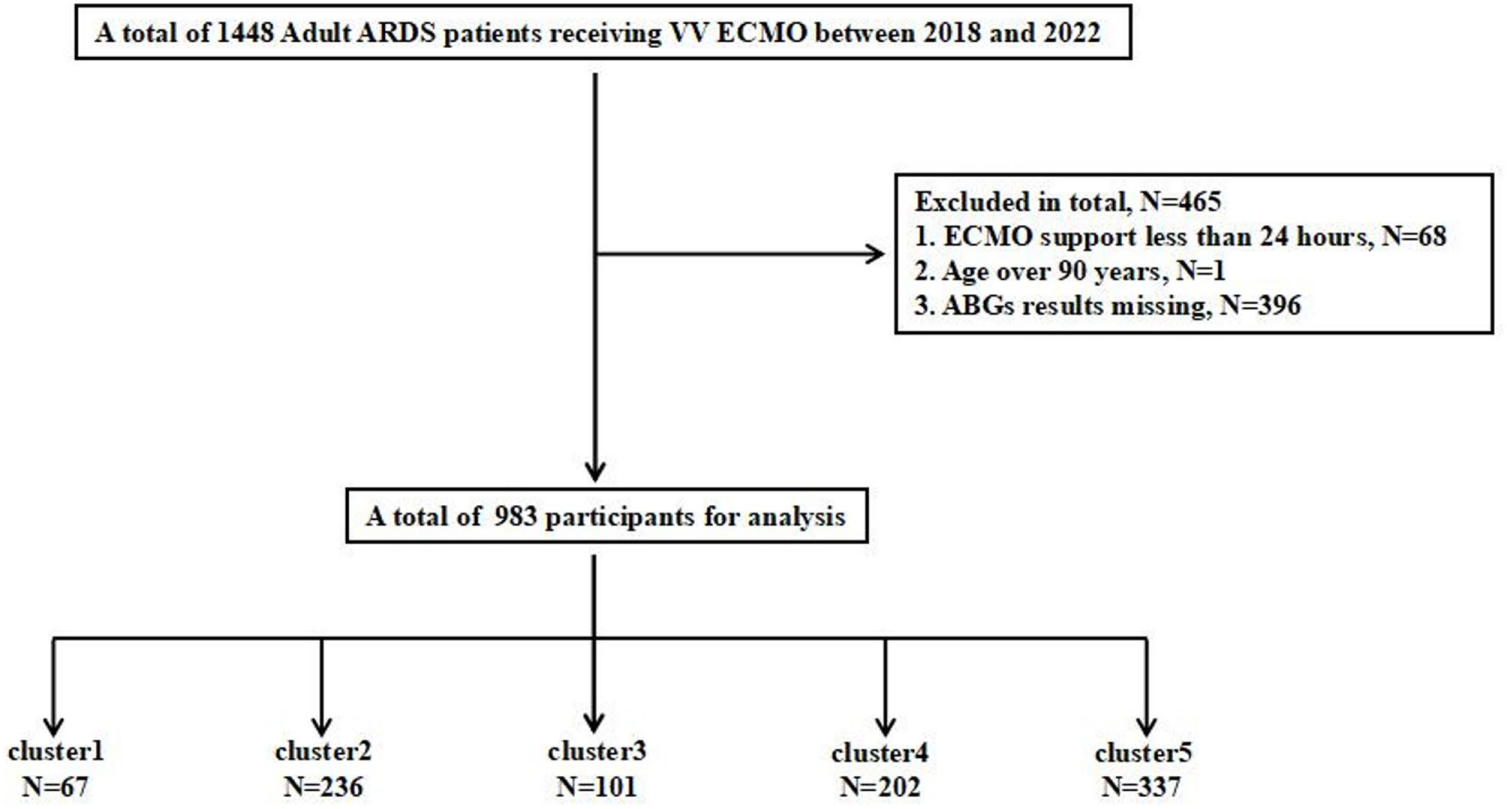
Flowchart of the study population; ABG: arterial blood gas; ECMO: extracorporeal membrane oxygenation; ABGs results missing including ABGs at 24 h after ECMO initiation and ABGs within 6 h before ECMO initiation

A total of 1,448 ARDS patients who received VV ECMO between 2018 and 2022 were initially identified. Of these, 465 were excluded due to ECMO duration of less than 24 h (*n* = 68), age over 90 years (*n* = 1), or missing ABG data (*n* = 396). Ultimately, 983 patients were included in the final analysis (Fig. 1). The mean age of the cohort was 52.41 ± 15.45 years, and 72.43% were male. The overall incidence of neurological complications was 2.95%, including seizures (0.1%), ischemic stroke (0.51%), intracranial hemorrhage (2.14%), and brain death (0.2%). The in-hospital mortality rate was 55.54% (Table 1).

**Table 1:** Characteristics and outcomes of different clusters

Variables	Total (*n* = 983)	Cluster1 (*n* = 67)	Cluster2 (*n* = 236)	Cluster3 (*n* = 101)	Cluster4 (*n* = 202)	Cluster5 (*n* = 377)	*P* value
ABG changes before and after ECMO initiation							
Abs ΔCO_2_, mmHg	-7.00 (-20.85, 2.00)	-50.00 (-63.80, -40.00)	5.00 (0.70, 10.00)	-5.00 (-12.30, 2.00)	-27.40 (-33.73, -22.00)	-5.60 (-11.80, -1.00)	< 0.001
Rel ΔCO_2_	-0.16 (-0.35,0.05)	-0.58 (-0.62, -0.53)	0.15 (0.02,0.30)	-0.10 (-0.25,0.08)	0.41 (-0.48, -0.35)	0.13 (-0.23, -0.02)	< 0.001
AbsΔpH	0.10 (0.01, 0.19)	0.34 (0.30, 0.40)	-0.04 (-0.09, -0.01)	0.28 (0.23, 0.34)	0.17 (0.13, 0.23)	0.08 (0.04, 0.12)	< 0.001
RelΔpH	0.01 (0.00, 0.03)	0.05 (0.04, 0.06)	-0.01 (-0.01, -0.00)	0.04 (0.03, 0.05)	0.02 (0.02, 0.03)	0.01 (0.01, 0.02)	< 0.001
ABGs within 6 h before ECMO initiation							
pH	7.32 (7.21, 7.41)	7.10 (7.01, 7.17)	7.45 (7.40, 7.49)	7.15 (7.11, 7.22)	7.23 (7.19, 7.29)	7.35 (7.29, 7.39)	< 0.001
PaCO_2_, mmHg	46.00 (36.35, 60.00)	92.00 (73.60, 111.00)	34.35 (29.95, 40.00)	45.00 (32.00, 50.00)	67.00 (59.00, 74.55)	45.00 (39.00, 51.00)	< 0.001
PaO_2_, mmHg	59.00 (50.00, 71.10)	68.00 (51.00, 81.85)	59.00 (49.30, 71.30)	55.00 (45.10, 67.80)	59.00 (50.00, 69.85)	59.00 (50.00, 70.00)	0.034
HCO_3_^−^, mmol/L	23.20 (19.00, 28.00)	26.00 (19.20, 31.10)	24.00 (20.70, 28.00)	16.00 (14.00, 20.00)	23.60 (19.00, 29.18)	23.60 (20.10, 27.30)	< 0.001
ABGs at 24 h after ECMO initiation							
pH	7.42 (7.36, 7.46)	7.44 (7.39, 7.50)	7.39 (7.35, 7.44)	7.45 (7.40, 7.48)	7.41 (7.36, 7.47)	7.43 (7.38, 7.47)	< 0.001
PaCO_2_, mmHg	39.00 (34.95, 44.00)	37.00 (33.00, 43.25)	40.70 (35.22, 46.00)	37.50 (32.30, 41.90)	38.95 (34.45, 43.88)	39.00 (35.00, 43.00)	< 0.001
PaO_2_, mmHg	85.00 (71.00, 107.15)	93.00 (80.20, 115.00)	83.20 (68.55, 104.00)	94.00 (73.00, 128.00)	84.75 (70.00, 100.00)	82.00 (72.00, 105.00)	0.009
HCO_3_^−^, mmol/L	25.00 (22.30, 28.00)	25.00 (22.45, 29.60)	24.80 (22.00, 27.40)	25.00 (22.70, 28.10)	24.70 (22.40, 28.15)	25.00 (22.80, 28.00)	0.31
Type of respiratory failure							< 0.001
Non-hypercapnic	571 (58.09%)	0 (0.00%)	224 (94.92%)	74 (73.27%)	7 (3.47%)	266 (70.56%)	
Hypercapnic	412 (41.91%)	67 (100.00%)	12 (5.08%)	27 (26.73%)	195 (96.53%)	111 (29.44%)	
Baseline characteristics							
Age, years	52.41 ± 15.45	54.42 ± 17.33	52.12 ± 15.78	51.60 ± 15.34	52.19 ± 14.79	52.56 ± 15.30	0.815
Sex, male	712 (72.43%)	53 (79.10%)	167 (70.76%)	77 (76.24%)	160 (79.21%)	255 (67.64%)	0.021
BMI, kg/m^2^	25.36 ± 20.99	24.69 ± 5.20	23.77 ± 3.82	31.86 ± 56.54	24.14 ± 4.14	25.38 ± 16.25	0.019
Pre-ECMO SOFA score	11.00 (8.00, 13.50)	11.00 (9.00, 14.00)	12.00 (8.00, 14.00)	13.00 (9.00, 15.00)	11.00 (8.00, 13.00)	11.00 (8.00, 13.00)	0.006
Duration of IMV before ECMO, hours	29.40 (6.58, 84.12)	42.33 (10.79, 118.12)	30.33 (6.00, 72.00)	21.32 (3.17, 63.00)	45.30 (11.53, 109.00)	25.42 (6.00, 81.87)	< 0.001
Comorbidities							
Chronic Cardiovascular Diseases	347 (35.30%)	28 (41.79%)	93 (39.41%)	19 (18.81%)	65 (32.18%)	142 (37.67%)	0.002
Diabetes	180 (18.31%)	14 (20.90%)	51 (21.61%)	18 (17.82%)	31 (15.35%)	66 (17.51%)	0.496
Cerebrovascular Accidents	50 (5.09%)	6 (8.96%)	7 (2.97%)	3 (2.97%)	12 (5.94%)	22 (5.84%)	0.203
Chronic Respiratory Diseases	166 (16.89%)	17 (25.37%)	32 (13.56%)	10 (9.90%)	42 (20.79%)	65 (17.24%)	0.026
Chronic Kidney Diseases	58 (5.90%)	3 (4.48%)	19 (8.05%)	4 (3.96%)	10 (4.95%)	22 (5.84%)	0.521
Malignacies	55 (5.60%)	6 (8.96%)	13 (5.51%)	6 (5.94%)	13 (6.44%)	17 (4.51%)	0.632
Cirrhosis	11 (1.12%)	0 (0.00%)	4 (1.69%)	0 (0.00%)	2 (0.99%)	5 (1.33%)	0.816
Immunocompromised status	92 (9.36%)	6 (8.96%)	22 (9.32%)	3 (2.97%)	15 (7.43%)	46 (12.20%)	0.053
Outcomes							
Neurological complications	29 (2.95%)	8 (11.94%)	7 (2.97%)	4 (3.96%)	4 (1.98%)	6 (1.59%)	< 0.001
Intracranial hemorrhage	21 (2.14%)	6 (8.96%)	5 (2.12%)	4 (3.93%)	3 (1.49%)	3 (0.8%)	0.002
Ischemic stroke	5 (0.51%)	2 (2.99%)	2 (0.85%)	0 (0.00%)	1 (0.50%)	0 (0.00)	0.026
Seizures	1 (0.10%)	0 (0.00%)	0 (0.005%)	0 (0.00%)	0 (0.00%)	1 (0.50%)	1.000
Brain death	2 (0.20%)	0 (0.00%)	0 (0.00%)	0 (0.00%)	0 (0.00%)	2 (0.53%)	0.658
Duration of ECMO, hours	209.58 (131.48, 354.82)	250.92 (124.50,400.56)	211.29 (139.21,369.56)	194.42 (114.30,279.00)	201.75 (130.95,330.92)	212.00 (135.50,360.00)	0.349
Successfully weaning from ECMO	526 (53.51%)	31 (46.27%)	123 (52.12%)	60 (59.41%)	104 (51.49%)	208 (55.17%)	0.440
In-Hospital mortality	543 (55.24%)	41 (61.19%)	132 (55.93%)	53 (52.48%)	111 (54.95%)	206 (54.64%)	0.848

Data are reported as n (%) or median (interquartile range)

ABG: arterial blood gas; BMI: body mass index; MAP: mean arterial pressure; HR: heart rate; ECMO: extracorporeal membrane oxygenation; IMV: invasive mechanical ventilation; SOFA: sequential organ failure score; Non-hypercapnic: PaCO_2_ < 50mmHg, Hypercapnic: PaCO_2_ ≥ 50mmHg

### K-means clustering based on absolute changes in PaCO_2_ and pH

**Fig. 2:**
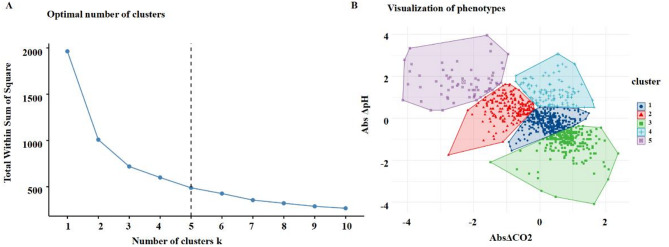
Clusters of the patients. (A) K-means clustering method for clustering the AbsΔCO_2_ and AbsΔpH; (B) The AbsΔCO_2_ and AbsΔpH clustering by K-means clustering

Patients were categorized into five clusters using K-means clustering based on the AbsΔCO_2_ and AbsΔpH. The optimal number of clusters (k = 5) was determined using cluster evaluation metrics **(**Fig. 2A**)**, and these clusters were used for subsequent analysis and interpretation **(**Fig. 2B**)**.

Jaccard index was employed to assess clustering stability, with values approaching 1 indicating enhanced cluster stability. Bootstrap resampling with 1000 replicates yielded Jaccard indices of 0.704, 0.756, 0.756, 0.809, and 0.816 across clusters, demonstrating satisfactory stability (all values > 0.7).

Cluster 1 (*n* = 67) demonstrated a substantial decrease in PaCO_2_, accompanied by a marked increase in pH and minimal change in bicarbonate levels. This cluster had the highest incidence of neurological complication among all 5 clusters. Median PaCO_2_ decreased from 90 mmHg pre-ECMO to 37 mmHg at 24 h post-ECMO, with an absolute reduction of 50 mmHg and a relative reduction of 58%. The pH increased from 7.10 to 7.44. Neurological complications occurred in 11.94% of patients, with an in-hospital mortality rate of 61.19%.

Cluster 3 (*n* = 101) showed a significant pH increase (from a median of 7.15 to 7.45), while PaCO_2_ decreased only slightly and not significantly (from 45 to 37.5 mmHg). A notable increase in bicarbonate levels suggested that the pH rise was mainly due to correction of metabolic acidosis rather than changes in PaCO_2_. Neurological complications occurred in 3.96% of patients, and the in-hospital mortality rate was 52.48%.

Cluster 4 (*n* = 202) also exhibited a reduction in PaCO_2_ and an increase in pH. However, the median pre-ECMO PaCO_2_ (67 mmHg) was lower than that of Cluster 1 (90 mmHg). The absolute reduction was 27.4 mmHg, corresponding to a relative reduction of 41%. Neurological complications occurred in 1.98% of patients, and the in-hospital mortality rate was 54.95%.

Cluster 2 (*n* = 236) and Cluster 5 (*n* = 377) showed relatively stable changes in PaCO_2_ and pH, with only minor fluctuations within physiological ranges. The incidence of neurological complications was 2.97% in Cluster 2 and 1.95% in Cluster 5. The in-hospital mortality rates were 55.93% and 54.64%, respectively.

Significant differences in the incidence of neurological complications were observed among the clusters (*P* < 0.001). Additional significant differences were noted between clusters in sex, BMI, pre-ECMO SOFA score, duration of IMV before ECMO, chronic cardiovascular disease and chronic respiratory disease (all *P* < 0.05) (Table 1).

Although the clustering was unsupervised, the five resulting groups aligned closely with common physiological patterns during ECMO initiation. Cluster 1 reflected rapid correction of respiratory acidosis (large PaCO_2_ drop with pH rise), while Cluster 3 indicated metabolic compensation (elevated bicarbonate and pH). Cluster 4 showed moderate changes in both parameters, suggesting mixed acid–base shifts. Clusters 2 and 5 exhibited only mild fluctuations, consistent with more stable states. These patterns support the clinical relevance of the clustering approach in identifying meaningful physiological subgroups.

### Neurological complications and in-hospital mortality outcomes across clusters

To determine whether the incidence of neurological complications and in-hospital mortality differed among clusters, a multivariate logistic regression model was constructed using Cluster 5 (the largest group with minimal changes in PaCO_2_ and pH) as the reference group (Table 2). Three models were developed to adjust for potential confounders. Patients in Cluster 1, characterized by the greatest changes in both AbsΔCO_2_ and AbsΔpH, exhibited a significantly higher risk of neurological complications compared to Cluster 5 across all three models (Model 1: OR = 8.38, 95% CI: 2.81–25.02, *P* < 0.001; Model 2: OR = 9.22, 95% CI: 3.05–27.90, *P* < 0.001; Model 3: OR = 11.69, 95% CI: 3.62–37.79, *P* < 0.001). However, in-hospital mortality did not significantly differ among clusters after adjusting for the same confounders (Table S1).

**Table 2:** Multivariate logistic regression for association between clusters and neurological complications

Variables	Model1		Model2		Model3	
	OR (95%CI)	*P*	OR (95%CI)	*P*	OR (95%CI)	*P*
Cluster						
5	1.00 (Reference)		1.00 (Reference)		1.00 (Reference)	
4	1.25 (0.35 ~ 4.48)	0.733	1.28 (0.35 ~ 4.61)	0.709	1.55 (0.41 ~ 5.90)	0.522
3	2.55 (0.71 ~ 9.21)	0.153	2.73 (0.75 ~ 9.99)	0.129	2.95 (0.76 ~ 11.45)	0.118
2	1.89 (0.63 ~ 5.69)	0.258	1.85 (0.61 ~ 5.59)	0.277	2.31 (0.72 ~ 7.45)	0.160
1	8.38 (2.81 ~ 25.02)	< 0.001	9.22 (3.05 ~ 27.90)	< 0.001	11.69 (3.62 ~ 37.79)	< 0.001

Model 1: Crude

Model 2: Adjusted for: Sex, Age, BMI, Pre-ECMO SOFA

Model 3: Adjusted for: Sex, Age, BMI, Pre-ECMO SOFA, Duration of IMV before ECMO (hours) and Comorbidities

Definition of abbreviations: BMI: body mass index; CI: confidence interval; Comorbidities including chronic cardiovascular diseases, chronic respiratory diseases, chronic kidney diseases, diabetes, malignancies, cerebrovascular accidents, immunocompromised status and cirrhosis. ECMO: extracorporeal membrane oxygenation; IMV: invasive mechanical ventilation. SOFA: sequential organ failure assessment

Taken together, the clustering analysis and regression analyses indicated that a large reduction in PaCO_2_ during the early phase of VV ECMO was significantly associated with an increased risk of neurological complications in ARDS patients. In contrast, pH changes alone, particularly those related to metabolic correction, were not independently associated with neurological risk. These findings highlight PaCO_2_ fluctuation as the primary gas exchange parameter of concern during the early management of VV ECMO-supported ARDS patients.

### Assessment of potentially safe PaCO_2_ reduction during early VV ECMO support

**Fig. 3:**
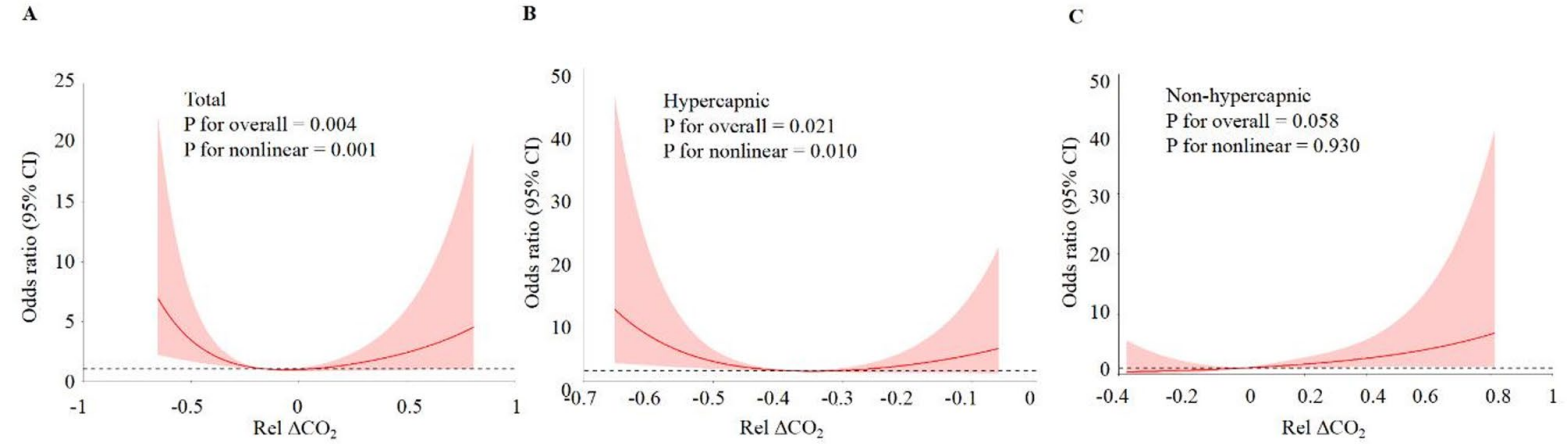
Cubic model of the association between Rel ΔCO_2_ and Neurological Complications in Different Respiratory Failure Subgroups. (A) All patients; (B) Hypercapnic(PaCO_2_ ≥ 50mmHg) ; (C) Non-hypercapnic (PaCO_2_ < 50mmHg)

Given the observed association between RelΔCO_2_ and neurological complications, we further investigated a potentially safe range of PaCO_2_ reduction during the early phase of VV ECMO. Restricted cubic spline regression in the full cohort (Fig. 3A) revealed a non-linear relationship between RelΔCO_2_ and the risk of neurological complications (*P* for nonlinear = 0.001). The risk increased with both excessive reductions and minimal changes in PaCO_2_, forming a U-shaped association.

Since hypercapnic patients were more frequently associated with a rapid decline in PaCO_2_ after ECMO initiation, patients were stratified by pre-ECMO PaCO_2_ level (*hypercapnic* or *non-hypercapnic*). Patient characteristics for both subgroups were presented in Table S2. Among patients in hypercapnic group, the spline model again showed a statistically significant non-linear relationship between RelΔCO_2_ and neurological complications (Fig. 3B). In contrast, no significant association was observed in non-hypercapnic group (Fig. 3C).

Based on the 25th and 75th percentiles of RelΔCO_2_, patients in hypercapnic group were categorized into three subgroups. Multivariable logistic regression revealed that patients with a PaCO_2_ reduction > 49% or < 27% had significantly higher risk of neurological complications compared to those in the middle range, after adjusting for relevant covariates (Table 3). These findings suggest that, in ARDS patients with hypercapnia (PaCO_2_ ≥ 50mmHg) receiving VV ECMO, a moderate reduction in PaCO_2_ (between 27% and 49%) within the first 24 h may be associated with a lower risk of neurological complications. However, since this threshold was derived from retrospective, data-driven analysis, it should be interpreted as exploratory rather than definitive.

**Table 3:** Multivariate logistic regression for association between different RelΔCO_2_ and neurological complications in hypercapnic group

Variables	Model 1		Model 2		Model 3	
	OR (95%CI)	*P*	OR (95%CI)	*P*	OR (95%CI)	*P*
RelΔCO_2_ in Hypercapnic group						
Group 1	1.00 (Reference)		1.00 (Reference)		1.00 (Reference)	
Group 2	4.39 (1.29 ~ 14.93)	0.018	4.47 (1.30 ~ 15.35)	0.017	4.53 (1.30 ~ 15.80)	0.018
Group 0	3.72 (1.06 ~ 13.01)	0.040	3.52 (0.99 ~ 12.53)	0.052	2.84 (0.76 ~ 10.57)	0.120

Model 1: Crude

Model 2: Adjusted for Sex, Age, BMI, Pre-ECMO SOFA

Model 3: Adjusted for Sex, Age, BMI, Pre-ECMO SOFA, Duration of IMV before ECMO (hours) and Comorbidities

Definition of abbreviations: BMI: body mass index; CI: confidence interval; Comorbidities including chronic cardiovascular diseases, chronic respiratory diseases, chronic kidney diseases, diabetes, malignancies, cerebrovascular accidents, immunocompromised status and cirrhosis. ECMO: extracorporeal membrane oxygenation; IMV: invasive mechanical ventilation. SOFA: sequential organ failure assessment

Patients in hypercapnic group were divided into three sub-groups according to the 25% and 75% quantiles of RelΔCO_2_. Group 0: RleΔCO_2_ >-27% (*n* = 103); Group 1: -49% ≤ RelΔCO_2_ ≤ -27% (*n* = 208); Group 2: RelΔCO_2_ ≤ -49% (*n* = 101)

## Discussion

In this multicenter, retrospective cohort study, we investigated the association between early fluctuations in PaCO_2_ and pH following VV ECMO initiation and the risk of neurological complications in patients with ARDS. Our findings suggest that large reductions in PaCO_2_ within the first 24 h of ECMO initiation were significantly associated with an increased risk of neurological complications. In contrast, changes in pH, particularly those resulting from metabolic acidosis correction, were not independently linked to increased neurological risk. These results support the hypothesis that PaCO_2_ fluctuation, rather than pH changes, is a more critical factor contributing to neurological injury during the early phase of VV ECMO support in ARDS.

The clustering analysis, although exploratory and data-driven, identified a high-risk subgroup (Cluster 1) characterized by the largest PaCO_2_ reductions (median 50 mmHg; relative reduction of 58%), which exhibited the highest incidence of neurological complications (11.94%). This finding aligns with previous reports by Luyt et al. [14]. and Cavayas et al. [8] and Hunsicker et al. [17], both of which demonstrated that excessive early reductions in PaCO_2_ were associated with increased risks of intracranial hemorrhage and other neurological events in ECMO-supported patients. Additional studies also emphasized the importance of gradual and controlled PaCO_2_ removal during ECMO initiation [18, 25]. In contrast, Yu et al. [20] did not report a statistically significant association between early PaCO_2_ changes and acute brain injury. However, the limited statistical power of that study, partly due to missing pre-ECMO ABG data in nearly 25% of participants and its single-center design, may have contributed to this discrepancy.

To further explore the relationship, we performed a restricted cubic spline analysis. A non-linear, U-shaped relationship between RelΔCO_2_ and neurological complications was observed, with increased risk at both minimal change and excessive reduction. Among patients with hypercapnia (pre-ECMO PaCO_2_ ≥ 50 mmHg), this non-linear relationship was particularly pronounced. Stratified logistic regression revealed that a moderate reduction in PaCO_2_ (27–49%) was associated with the lowest risk of neurological complications. Excessive PaCO_2_ reduction may have induced cerebral vasoconstriction, decreased cerebral perfusion pressure, and impaired oxygenation, all of which are known contributors to neurological injury [5, 21, 24–26]. Although the U-shaped curve suggests that an insufficient reduction may also be associated with harm, there is currently no direct evidence supporting a causal link between persistent hypercapnia and neurological complications. Persistent high PaCO_2_ level may potentially be due to imbalances between reduced ventilatory support (e.g., lower tidal volume and respiratory rate) and sweep gas flow after ECMO initiation. These observations underscore the need for prospective studies to better understand the physiological implications of both minimal and excessive PaCO_2_ changes. While our findings point to a potentially safer range of PaCO_2_ reduction, this threshold was derived from retrospective, data-driven analysis and should be interpreted as exploratory rather than prescriptive.

Although PaCO_2_ and pH are generally interrelated, pH can also be influenced by bicarbonate levels. The relative contribution of PaCO_2_ and pH changes to neurological complications remains unclear. Patients in Cluster 3, in whom pH increased primarily due to correction of metabolic acidosis, experienced a relatively low incidence of neurological complications (3.96%), suggesting that pH may have played a less significant role than PaCO_2_ fluctuations in driving neurological injury. Nonetheless, while pH shifts, particularly alkalosis or acidosis correction, can affect cerebrovascular blood flow and potentially exacerbate the effects of PaCO_2_ fluctuations [27, 28]. While our findings support PaCO_2_ fluctuation as the primary determinant of neurological complications, the potential influence of pH should not be disregarded.

We intentionally restricted our analysis to patients receiving VV ECMO in order to minimize confounding effects from circulatory support variables associated with VA ECMO, such as hemodynamic instability and differential oxygen delivery. Prior studies have shown that VA ECMO is linked to higher rates of neurological complications, likely due to the combined effects of impaired perfusion, altered gas exchange, and increased embolic risk [6, 29]. By focusing solely on VV ECMO, our analysis enabled a more precise assessment of the respiratory contribution to neurological outcomes.

Despite strengths of a large, multicenter registry and rigorous statistical methods, several limitations must be acknowledged. First, the retrospective design inherently introduced selection bias and limits the ability to draw causal inferences. Although we adjusted for several key confounders, a major methodological limitation is the absence of platelet count, which is a well-established independent risk factor for intracranial hemorrhage during ECMO support. Unfortunately, platelet values were not available in the CSECLS registry and could not be included in the analyses. Other critical clinical variables such as anticoagulation strategy and levels, ventilator settings, and ECMO parameters (e.g., sweep gas flow) were also unavailable, further restricting comprehensive adjustment for confounding. Unmeasured center-level practices may also have influenced outcomes. Additionally, our analysis focused exclusively on neurological complications and did not account for other ECMO-related adverse events—such as bleeding, thrombosis, or infection—which may interact with neurological risk or confound clinical outcomes. Second, the observed incidence of neurological complications in our cohort (2.95%) was lower than that reported in similar studies, such as the multicenter analysis by Cavayas et al., which documented a 6.9% incidence using the ELSO database [9]. This discrepancy may reflect differences in complication definitions, neuro-monitoring intensity, or underreporting in our registry, potentially underestimating the true burden of neurological injury. Third, although our primary outcome was based on clinically validated definitions within the CSECLS registry, we could not distinguish between subtypes of intracranial hemorrhage (e.g., subarachnoid, subdural, or intraparenchymal) due to the absence of detailed neuroimaging data. This limits insight into potential differences in etiology and associations with gas exchange variables. Furthermore, in ECMO-supported patients with coagulopathy or vascular fragility, differentiating between primary hemorrhage and secondary hemorrhagic transformation of ischemic injury is often challenging. The lack of temporal and imaging data in our dataset limits nuanced interpretation. Fourth, we were unable to determine the exact timing of neurological events relative to ECMO initiation due to limitations in the registry data. As a result, we could not establish a clear temporal link between early PaCO_2_ changes and the onset of complications, particularly for those that may have occurred several days into ECMO support. This impedes causal inference and highlights the need for future studies with real-time neurological monitoring and event logging. Fifth, in critically ill patients undergoing VV ECMO, early mortality may act as a competing risk, potentially preventing the detection or documentation of neurological complications that occur later during the ECMO course. However, due to limitations in the CSECLS registry, the precise timing of neurological complication onset is not recorded, precluding the use of formal time-to-event or competing risk models in this study. Furthermore, although pH changes were not independently associated with neurological complications in our analysis, their interaction with PaCO_2_ fluctuations warrants further study. Lastly, while our spline analysis suggested a non-linear (U-shaped) relationship between PaCO_2_ change and neurological complications, the physiological interpretation of minimal CO_2_ reduction remains unclear. Therefore, the identified thresholds should be interpreted as exploratory and require prospective validation. Future research should incorporate detailed physiological and neuroimaging data, real-time event tracking, and prospective cohort designs to refine risk stratification and optimize ECMO management strategies aimed at minimizing neurological complications VV ECMO supported ARDS patients.

## Conclusion

In conclusion, excessive reductions in PaCO_2_ during the early phase of VV ECMO initiation, rather than increases in pH, were associated with a higher incidence of neurological complications in patients with ARDS. Our findings suggest that avoiding PaCO_2_ reductions exceeding 50% from baseline may help reduce the risk of neurological injury in this population. These results underscore the importance of carefully managing ECMO settings to prevent abrupt PaCO_2_ shifts and optimize patient outcomes. Further research is warranted to refine ECMO management strategies and to develop individualized approaches for optimizing gas exchange while minimizing neurological risk in patients ARDS supported by VV ECMO.

## Supplementary Information

Below is the link to the electronic supplementary material.

Supplementary Material 1. Table S1. Multivariate Logistic Regression for Association between Different Clusters and In-hospital Mortality.

Supplementary Material 2. Table S2. Characteristics and Outcomes of Different Sub-groups in Hypercapnic Group.

## Data Availability

All data generated or analyzed during this study are included in this published article.

## References

[1] Matthay MA, Zemans RL, Zimmerman GA, Arabi YM, Beitler JR, Mercat A, et al. Acute respiratory distress syndrome. Nat Rev Dis Primer. 2019;5:1–22. 10.1038/s41572-019-0069-0 PMC6709677 30872586

[2] Bellani G, Laffey JG, Pham T, Fan E, Brochard L, Esteban A, et al. Epidemiology, patterns of care, and mortality for patients with acute respiratory distress syndrome in intensive care units in 50 countries. JAMA. 2016;315:788–800. 26903337 10.1001/jama.2016.0291

[3] Sadana D, Kaur S, Sankaramangalam K, Saini I, Banerjee K, Siuba M, et al. Mortality associated with acute respiratory distress syndrome, 2009–2019: a systematic review and meta-analysis. Crit Care Resusc J Australas Acad Crit Care Med. 2022;24:341–51. 10.51893/2022.4.OA4 PMC10692616 38047005

[4] Bromley SE, Shakery K, Vora P, Atabaki A, Reimer T, McDermott L, et al. Understanding causes of death in patients with acute respiratory distress syndrome: A narrative review. Crit Care Explor. 2024;6:e1147. 39172623 10.1097/CCE.0000000000001147 PMC11343544

[5] Tonna JE, Abrams D, Brodie D, Greenwood JC, Mateo-Sidron JAR, Usman A, et al. Management of adult patients supported with venovenous extracorporeal membrane oxygenation (VV ECMO): guideline from the extracorporeal life support organization (ELSO). ASAIO J Am Soc Artif Intern Organs 1992. 2021;67:601–10. 10.1097/MAT.0000000000001432 PMC8315725 33965970

[6] Pisano DV, Ortoleva JP, Wieruszewski PM. Short-Term neurologic complications in patients undergoing extracorporeal membrane oxygenation support: A review on Pathophysiology, Incidence, risk Factors, and outcomes. Pulm Ther. 2024;10:267–78. 38937418 10.1007/s41030-024-00265-z PMC11339018

[7] Kim K, Leem AY, Kim SY, Chung KS, Park MS, Kim YS, et al. Complications related to extracorporeal membrane oxygenation support as a Bridge to lung transplantation and their clinical significance. Heart Lung J Crit Care. 2022;56:148–53. 10.1016/j.hrtlng.2022.07.008 35908349

[8] Zhang H, Xu J, Yang X, Zou X, Shu H, Liu Z, et al. Narrative review of neurologic complications in adults on ECMO: Prevalence, Risks, Outcomes, and prevention strategies. Front Med. 2021;8:713333. 10.3389/fmed.2021.713333 PMC8513760 34660625

[9] Cavayas YA, Munshi L, Del Sorbo L, Fan E. The early change in PaCO2 after extracorporeal membrane oxygenation initiation is associated with neurological complications. Am J Respir Crit Care Med. 2020;201:1525–35. 32251606 10.1164/rccm.202001-0023OC

[10] Cho S-M, Canner J, Caturegli G, Choi CW, Etchill E, Giuliano K, et al. Risk factors of ischemic and hemorrhagic strokes during venovenous extracorporeal membrane oxygenation: analysis of data from the extracorporeal life support organization registry. Crit Care Med. 2021;49:91–101. 33148951 10.1097/CCM.0000000000004707 PMC9513801

[11] Shou BL, Ong CS, Zhou AL, Al-Kawaz MN, Etchill E, Giuliano K, et al. Arterial carbon dioxide and acute brain injury in venoarterial extracorporeal membrane oxygenation. ASAIO J Am Soc Artif Intern Organs 1992. 2022;68:1501–7. 10.1097/MAT.0000000000001699 PMC9477972 35671442

[12] Shigemura M, Lecuona E, Sznajder JI. Effects of hypercapnia on the lung. J Physiol. 2017;595:2431–7. 28044311 10.1113/JP273781 PMC5390878

[13] Nin N, Angulo M, Briva A. Effects of hypercapnia in acute respiratory distress syndrome. Ann Transl Med. 2018;6:37. 29430454 10.21037/atm.2018.01.09 PMC5799147

[14] Luyt C-E, Bréchot N, Demondion P, Jovanovic T, Hékimian G, Lebreton G, et al. Brain injury during venovenous extracorporeal membrane oxygenation. Intensive Care Med. 2016;42:897–907. 27007107 10.1007/s00134-016-4318-3

[15] Kahl U, Yu Y, Nierhaus A, Frings D, Sensen B, Daubmann A, et al. Cerebrovascular autoregulation and arterial carbon dioxide in patients with acute respiratory distress syndrome: a prospective observational cohort study. Ann Intensive Care. 2021;11:47. 33725209 10.1186/s13613-021-00831-7 PMC7962086

[16] Ficial B, Vasques F, Zhang J, Whebell S, Slattery M, Lamas T, et al. Physiological basis of extracorporeal membrane oxygenation and extracorporeal carbon dioxide removal in respiratory failure. Membranes. 2021;11:225. 33810130 10.3390/membranes11030225 PMC8004966

[17] Hunsicker O, Beck L, Krannich A, Finger T, Prinz V, Spies C, et al. Timing, Outcome, and risk factors of intracranial hemorrhage in acute respiratory distress syndrome patients during venovenous extracorporeal membrane oxygenation. Crit Care Med. 2021;49:e120–9. 33323749 10.1097/CCM.0000000000004762

[18] Shah N, Li X, Shanmugham P, Fan E, Thiagarajan RR, Venkataraman R, et al. Early changes in arterial partial pressure of carbon dioxide and blood pressure after starting extracorporeal membrane oxygenation in children: extracorporeal life support organization database study of neurologic complications. Pediatr Crit Care Med J Soc Crit Care Med World Fed Pediatr Intensive Crit Care Soc. 2023;24:541–50. 10.1097/PCC.0000000000003216 36877009

[19] Le Guennec L, Cholet C, Huang F, Schmidt M, Bréchot N, Hékimian G, et al. Ischemic and hemorrhagic brain injury during venoarterial-extracorporeal membrane oxygenation. Ann Intensive Care. 2018;8:129. 30570687 10.1186/s13613-018-0475-6 PMC6301905

[20] Y Y. Association of early changes in arterial carbon dioxide with acute brain injury in adult patients with extracorporeal membrane oxygenation: A ten-year retrospective study in a German tertiary care hospital. J Crit Care. 2024;84:154880. 39024824 10.1016/j.jcrc.2024.154880

[21] Shoskes A, Migdady I, Rice C, Hassett C, Deshpande A, Price C, et al. Brain injury is more common in venoarterial extracorporeal membrane oxygenation than venovenous extracorporeal membrane oxygenation: A systematic review and Meta-Analysis. Crit Care Med. 2020;48:1799–808. 33031150 10.1097/CCM.0000000000004618

[22] Ferguson ND, Fan E, Camporota L, Antonelli M, Anzueto A, Beale R, et al. The Berlin definition of ARDS: an expanded rationale, justification, and supplementary material. Intensive Care Med. 2012;38:1573–82. 22926653 10.1007/s00134-012-2682-1

[23] Hennig C. Dissolution point and isolation robustness: robustness criteria for general cluster analysis methods. J Multivar Anal. 2008;99:1154–76.

[24] Fernando SM, Brodie D, Barbaro RP, Agerstrand C, Badulak J, Bush EL, et al. Age and associated outcomes among patients receiving venovenous extracorporeal membrane oxygenation for acute respiratory failure: analysis of the extracorporeal life support organization registry. Intensive Care Med. 2024;50:395–405. 38376515 10.1007/s00134-024-07343-5

[25] Cho S-M, Gusdon AM. Assessing acute brain injury after rapid reduction of PaCO2 using plasma biomarkers in patients undergoing ECMO. Neurocrit Care. 2024;41:6–8. 38356080 10.1007/s12028-024-01944-0 PMC11414778

[26] Hajage D, Combes A, Guervilly C, Lebreton G, Mercat A, Pavot A, et al. Extracorporeal membrane oxygenation for severe acute respiratory distress syndrome associated with COVID-19: an emulated target trial analysis. Am J Respir Crit Care Med. 2022;206:281–94. 35533052 10.1164/rccm.202111-2495OC PMC9890253

[27] Yoon S, Zuccarello M, Rapoport RM. pCO2 and pH regulation of cerebral blood flow. Front Physiol. 2012;3:365. 23049512 10.3389/fphys.2012.00365 PMC3442265

[28] Palmer BF, Clegg DJ. Respiratory acidosis and respiratory alkalosis: core curriculum 2023. Am J Kidney Dis. 2023;82:347–59. 37341662 10.1053/j.ajkd.2023.02.004

[29] Su Y, Liu K, Zheng J-L, Li X, Zhu D-M, Zhang Y, et al. Hemodynamic monitoring in patients with venoarterial extracorporeal membrane oxygenation. Ann Transl Med. 2020;8:792. 32647717 10.21037/atm.2020.03.186 PMC7333156

