# Supplementary material for: Impact of early PaCO2 and pH fluctuations on neurological outcomes in ARDS patients receiving VV ECMO: a retrospective cohort study from the CSECLS registry
Source: Ann Intensive Care. 2025 Sep 25;15:143. doi: 10.1186/s13613-025-01570-9 (PMC12463811; doi:10.1186/s13613-025-01570-9)
Supplement: Supplementary file 1 — Supplementary Material 1. Table S1. Multivariate Logistic Regression for Association between Different Clusters and In-hospital Mortality. [file 13613_2025_1570_MOESM1_ESM.docx]

**Table S1. Multivariate Logistic Regression for Association between Different Clusters and In-hospital Mortality.**

| Variables | Model 1 | |  | Model 2 | |  | Model 3 | |
| --- | --- | --- | --- | --- | --- | --- | --- | --- |
|  | OR (95%CI) | *P* |  | OR (95%CI) | *P* |  | OR (95%CI) | *P* |
| Cluster |  |  |  |  |  |  |  |  |
| 5 | 1.00 (Reference) |  |  | 1.00 (Reference) |  |  | 1.00 (Reference) |  |
| 4 | 1.01 (0.72 ~ 1.43) | 0.943 |  | 1.02 (0.72 ~ 1.45) | 0.913 |  | 1.02 (0.71 ~ 1.46) | 0.911 |
| 3 | 0.92 (0.59 ~ 1.42) | 0.698 |  | 0.86 (0.55 ~ 1.36) | 0.530 |  | 0.90 (0.56 ~ 1.43) | 0.647 |
| 2 | 1.05 (0.76 ~ 1.46) | 0.755 |  | 1.04 (0.74 ~ 1.45) | 0.831 |  | 1.11 (0.79 ~ 1.57) | 0.551 |
| 1 | 1.31 (0.77 ~ 2.23) | 0.321 |  | 1.22 (0.71 ~ 2.12) | 0.472 |  | 1.21 (0.69 ~ 2.11) | 0.501 |
| Model1: Crude | | | | | | | | |
| Model2: Adjusted for: Sex, Age, BMI, Pre-ECMO SOFA | | | | | | | | |
| Model3: Adjusted for: Sex, Age, BMI, Pre-ECMO SOFA, Duration of IMV before ECMO (hours) and Comorbidities. | | | | | | | | |
| Definition of abbreviations: BMI: body mass index; CI= confidence interval; Comorbidities including chronic cardiovascular diseases, chronic respiratory diseases, chronic kidney diseases, diabetes, malignancies, cerebrovascular accidents, immunocompromised status and cirrhosis. ECMO: extracorporeal membrane oxygenation; IMV: invasive mechanical ventilation. SOFA: sequential organ failure assessment. | | | | | | | | |
