# Supplementary material for: Impact of early PaCO2 and pH fluctuations on neurological outcomes in ARDS patients receiving VV ECMO: a retrospective cohort study from the CSECLS registry
Source: Ann Intensive Care. 2025 Sep 25;15:143. doi: 10.1186/s13613-025-01570-9 (PMC12463811; doi:10.1186/s13613-025-01570-9)
Supplement: Supplementary file 2 — Supplementary Material 2. Table S2. Characteristics and Outcomes of Different Sub-groups in Hypercapnic Group. [file 13613_2025_1570_MOESM2_ESM.docx]

**Table S2. Characteristics and Outcomes of different Sub-groups in Hypercapnic Group**

| **Variables** | **Total (n = 412)** | **Group 0 (n =103)** | **Group 1 (n =208)** | **Group 2 (n =101)** | ***P* value** |
| --- | --- | --- | --- | --- | --- |
| **ABG changes before and after ECMO initiation** |  |  |  |  |  |
| AbsΔCO_2_, mmHg | -24.00  (-36.00, -15.00) | -24.00  (-30.40, -19.55) | -42.00  (-60.60, -37.50) | -11.10  (-13.00, -8.05) | <.001 |
| RelΔCO_2_ | -0.38  (-0.49, -0.27) | -0.38  (-0.43, -0.33) | -0.55  (-0.60, -0.52) | -0.20  (-0.24, -0.15) | <.001 |
| AbsΔpH | 0.16  (0.10, 0.26) | 0.17  (0.11, 0.24) | 0.26  (0.19, 0.34) | 0.11  (0.06, 0.14) | <.001 |
| RelΔpH | 0.02  (0.01, 0.04) | 0.02  (0.01, 0.03) | 0.04  (0.03, 0.05) | 0.02  (0.01, 0.02) | <.001 |
| **ABGs within 6 hours before ECMO initiation** |  |  |  |  |  |
| pH | 7.23 (7.17, 7.32) | 7.23 (7.17, 7.32) | 7.18 (7.08, 7.22) | 7.28 (7.22, 7.35) | <.001 |
| PaCO_2_, mmHg | 64.00  (55.63, 74.10) | 63.95  (56.67, 72.00) | 79.00  (69.50, 99.00) | 54.80  (51.05, 59.00) | <.001 |
| PaO_2_, mmHg | 60.00  (50.00, 73.08) | 58.50  (50.00, 72.62) | 62.00  (50.00, 78.00) | 60.00  (50.50, 70.50) | 0.290 |
| HCO_3_^-^, mmol/L | 24.85  (20.00, 29.82) | 23.95  (19.00, 29.30) | 26.00  (19.40, 31.80) | 25.30  (21.20, 29.15) | 0.467 |
| **ABGs at 24 hours after ECMO initiation** |  |  |  |  |  |
| pH | 7.41 (7.36, 7.46) | 7.41 (7.36, 7.46) | 7.43 (7.38, 7.49) | 7.40 (7.35, 7.45) | 0.004 |
| PaCO_2_, mmHg | 40.00  (36.00, 45.00) | 40.00  (36.80, 44.32) | 35.00  (32.00, 39.60) | 45.00  (41.80, 48.85) | <.001 |
| PaO_2_, mmHg | 86.00  (71.00, 106.25) | 84.45  (70.00, 100.00) | 91.10  (75.00, 115.20) | 82.00  (71.00, 107.00) | 0.089 |
| HCO_3_^-^, mmol/L | 25.00  (22.60, 28.82) | 25.00  (23.00, 28.78) | 24.00  (21.30, 26.90) | 26.00  (23.60, 30.15) | 0.005 |
| **Baseline characteristics** |  |  |  |  |  |
| Age, years | 53.34 ± 15.70 | 53.82 ± 15.46 | 53.11 ± 15.57 | 52.60 ± 16.41 | 0.803 |
| Sex, Male | 307 (74.51) | 65 (63.11) | 163 (78.37) | 79 (78.22) | 0.009 |
| BMI, kg/m^2^ | 25.10 ± 15.48 | 24.22 ± 3.66 | 24.45 ± 4.70 | 27.51 ± 30.14 | 0.188 |
| Pre-ECMO SOFA score | 11.00  (8.00, 13.00) | 11.00  (8.00, 13.25) | 11.00  (9.00, 14.00) | 12.00  (8.00, 13.00) | 0.604 |
| Duration of IMV before ECMO, hours | 39.17  (10.75, 107.66) | 41.00  (11.27, 106.25) | 42.00  (11.10, 155.00) | 27.15  (9.25, 95.82) | 0.469 |
| **Comorbidities** |  |  |  |  |  |
| Chronic Cardiovascular Diseases | 144 (34.95) | 32 (31.07) | 72 (34.62) | 40 (39.60) | 0.437 |
| Diabetes | 68 (16.50) | 12 (11.65) | 36 (17.31) | 20 (19.80) | 0.265 |
| Cerebrovascular Accidents | 28 (6.80) | 5 (4.85) | 16 (7.69) | 7 (6.93) | 0.644 |
| Chronic Respiratory Diseases | 88 (21.36) | 24 (23.30) | 40 (19.23) | 24 (23.76) | 0.566 |
| Chronic Kidney Diseases | 18 (4.37) | 3 (2.91) | 10 (4.81) | 5 (4.95) | 0.776 |
| Malignancies | 25 (6.07) | 4 (3.88) | 14 (6.73) | 7 (6.93) | 0.561 |
| Cirrhosis | 2 (0.49) | 0 (0.00) | 2 (0.96) | 0 (0.00) | 1.000 |
| Immunocompromised status | 35 (8.50) | 8 (7.77) | 18 (8.65) | 9 (8.91) | 0.951 |
| **Outcomes** |  |  |  |  |  |
| Neurological complications | 19 (4.61) | 7 (6.80) | 4 (1.92) | 8 (7.92) | 0.021 |
| Intracranial hemorrhage | 15 (3.64%) | 6 (5.83%） | 3 (1.44%） | 6 (5.94%) | 0.047 |
| Ischemic stroke | 3 (0.73%) | 0 (0.00%) | 1 (0.48%) | 2 (1.98%) | 0.211 |
| Seizures | 1 (0.24%) | 1 (0.97%) | 0 (0.00%) | 0 (0.00%) | 0.495 |
| Brain death | 0 (0.00%） | 0 (0.00%） | 0 (0.00%) | 0 (0.00%） | - |
| Duration of ECMO，hours | 212.26  (125.56, 351.91) | 236.17  (144.00, 414.85) | 200.08  (131.62, 316.72) | 212.67  (91.50, 361.33) | 0.303 |
| Successfully ECMO weaning from ECMO | 204 (49.51%) | 47 (46.53%) | 56 (54.37%) | 101 (48.56%) | 0.495 |
| Hospital mortality | 235 (57.04%) | 55 (53.40%) | 118 (56.73%) | 62 (61.39%) | 0.511 |

Data are reported as n (%) or median [interquartile range]. ABG: arterial blood gas; BMI: body mass index; ECMO: extracorporeal membrane oxygenation; IMV: invasivse mechanical ventilation; SOFA: sequential organ failure score. Decreasing sub-groups were divided into three cluster according to the 25% and 75% quantiles of RelΔCO_2_. Group 0: RelΔCO_2_ > -27%; Group 1: -49% ≤ RelΔCO_2_ ≤ -27% (n=208); Group 2: RelΔCO_2_ ≤ -49% (n=101).
